# Development of communication tool for resident‐ and family‐led care discussions in long‐term care through patient and family engagement

**DOI:** 10.1111/opn.12429

**Published:** 2021-10-07

**Authors:** Lisa Cranley, Gajan Sivakumaran, Shoshana Helfenbaum, Daniel Galessiere, Raquel Meyer, Wendy Duggleby, Linda McGillis Hall, Katherine S. McGilton

**Affiliations:** ^1^ Lawrence S. Bloomberg Faculty of Nursing University of Toronto Toronto ON Canada; ^2^ Leslie Dan Faculty of Pharmacy University of Toronto Toronto ON Canada; ^3^ Ontario Centres for Learning, Research and Innovation in Long‐Term Care at Baycrest Toronto ON Canada; ^4^ Faculty of Nursing University of Alberta Edmonton AB Canada; ^5^ KITE ‐ Toronto Rehabilitation Institute University Health Network Toronto ON Canada

**Keywords:** care planning, communication tool, interviews, long‐term care, patient engagement

## Abstract

**Background:**

Effective communication between residents (older adults), families, and the healthcare team supports person‐centred care. However, communication breakdowns can occur that can impact care and outcomes. The aim of this paper is to describe a feedback approach to developing a communication tool for residents and families to guide information sharing during care discussions with the healthcare team in long‐term care.

**Methods:**

Development of the communication tool included consultation with key stakeholders for their feedback and input. Following initial development of the tool template by our research team, we invited feedback from our study collaborators. Next, individual interviews and a focus group were conducted with family members, followed by individual interviews with selected residents from two long‐term care homes in Ontario, Canada. Participants were asked to provide input and feedback on the tool's content and usability and to share ideas for improving the tool. Content analysis was used to analyse the interview data.

**Results:**

Feedback from residents and family included suggestions to enhance the tool's content and use of plain language, and suggestions for potential application of the tool.

**Conclusion:**

Feedback highlighted the value of engaging residents and family members in the development of a communication tool. The communication tool offers a structured format to support participation of residents and families in information sharing for care discussions with the healthcare team.

**What does this research add to existing knowledge in gerontology?**

- This study describes a feedback approach to the development of a communication tool that engaged older adults (residents) in long‐term care and their families.
- This study addresses calls for a shift in focus from communication tools developed for healthcare professionals to those developed specifically for (and with) patients and families to guide care conversations.
- Consulting with residents and families during the development of a communication tool intended for their use can ensure the tool is appropriate and relevant.

**What are the implications of this new knowledge for nursing care with older people?**

- The communication tool provides residents and families with a structured way to lead care conversations and share information with teams.
- It is important to partner with residents and families when developing tailored nursing care interventions.

**How could the findings be used to influence policy or practice or research or education?**

- Engaging residents and families in research is a meaningful activity as they are experts of their experience.
- Further research is needed to examine the broader application of communication tools for residents and families to use to guide or lead care conversations with healthcare teams in long‐term care.
- Future research should examine the impact of end‐user input and feedback processes on the uptake of tools and interventions for quality improvement.

## 1 BACKGROUND

The presence of good communication in healthcare is the foundation for safe, high‐quality care (Institute of Medicine, 2001). However, communication breakdown in healthcare settings is pervasive (Institute of Medicine, 2001). While ineffective communication sometimes exists among healthcare providers (Foronda et al., 2016), it is also present between healthcare providers and family members. For example, in the long‐term care (LTC) sector, poor communication exists between the healthcare team (hereafter care team—e.g. nurses, personal support workers, allied healthcare providers) and family members of residents because of miscommunication or lack of information exchange, which can lead to conflicts (Cranley et al., 2020; Haesler et al., 2006; Pillemer et al., 2003). Personal support workers (unregulated providers also referred to as care aides or nursing assistants) are the largest workforce in LTC, and they are often excluded from team communication and decision‐making (Caspar et al., 2016). They typically do not join team conferences (e.g. due to lack of time during their shift) (Caspar et al., 2016) and are often directed by their administrators to refer family members to the nurses if they have concerns (McGilton et al., 2008). These structural issues make it difficult for them to communicate up‐to‐date information to families.

Families play a key role in the quality of resident care because they have knowledge of the resident's life history, needs, and preferences and act as advocates to foster person‐centred care (Kolanowski et al., 2013; Robison et al., 2007). The relationship between family and the care team in LTC is ideally a negotiated partnership (Haesler et al., 2006; Jang, 2020). Managing conflicts and encouraging family involvement and inclusion in care can facilitate effective communication and strengthen relationships between families and the care team, which contribute to the well‐being of residents and family (Barken & Lowndes, 2018; Puurveen et al., 2018; Robison et al., 2007; Ryan & Scullion, 2000).

Effective communication between the care team and residents with cognitive and/or sensory impairment can be challenging as residents especially with dementia may have difficulties with expressing their needs, asking for help, or understanding others (Christenson et al., 2011; McGilton et al., 2017). Yet, it is essential to optimise communication and interactions between the care team and residents to support person‐centred care and build partnered relationships and interpersonal connections (Banerjee & Rewegen, 2016; Kolanowski et al., 2013; McGilton & Boscart, 2007; Rockwell, 2012; Savundranayagam, 2013). Research has shown that residents value social interactions, relationships, and connectedness as these aspects contribute to their quality of life (O'Rourke et al., 2015). In our earlier study on resident, family, and nurses’ perceptions of shared decision‐making in LTC, findings highlighted a need for more proactive communication and information sharing among the care team, residents, and their family (Cranley et al., 2020). A systematic review found that formal communication among care teams in LTC homes (e.g. scheduled meetings) can have a positive impact on resident outcomes such as a reduction in the use of antipsychotics and restraints (Nazir et al., 2013).

Communication skills training programs have traditionally focused on improving healthcare providers skills (Berkhof et al., 2011; Cappi et al., 2019; Mata et al., 2021; Moore et al., 2018). There is a growing literature in LTC that focuses on communication skills training to improve team–family communication (Pillemer et al., 2003; Robison et al., 2007) and team–resident communication, particularly residents with dementia (Egan et al., 2010; Eggenberger et al., 2013; Franzmann et al., 2016; Machiels et al., 2017; McGilton et al., 2009, 2017; Passalacqua & Harwood, 2012; Vasse et al., 2010). These interventions often include didactic components such as video and discussion and/or role play (Eggenberger et al., 2013). Communication skills training has shown positive outcomes for family members and residents. For example, training has demonstrated improved family communication skills and decreased family–team conflict (Pillemer et al., 2003; Robison et al., 2007), increased family involvement in care (Robison et al., 2007), and has shown a positive effect on resident well‐being and quality of life (Eggenberger et al., 2013; McGilton et al., 2017).

Communication skills training can also include the use of structured communication tools such as SOAP (Subjective, Objective, Assessment, Plan), TJC‐CDPH (The Joint Commission Communication During Patient Handoff), and SBAR (Situation, Background, Assessment, Recommendation), which have largely been used in hospitals for patient handoff and transfer of patient care among healthcare professionals (Shahid & Thomas, 2018). Structured communication tools have been implemented to address communication breakdowns among clinicians and promote patient safety, by improving the efficiency and effectiveness of interprofessional communication (Muller et al., 2018; Shahid & Thomas, 2018). To the best of our knowledge, these tools have not been widely implemented in LTC homes.

There have been calls for a shift in focus from communication tools for healthcare providers to communication tools for patients and families (Lindberg et al., 2013). Communication tools have been advocated for use by patients and families with care teams, to empower them to participate in their care (Clochesy et al., 2015; Denham, 2008; Lindberg et al., 2013). The literature on improving communication has largely focused on healthcare providers, and the broader application of communication tools for patients and families has only begun to emerge. For example, structured communication tools have been developed to support patient communication with healthcare providers in acute care. For example, the Ask Me 3^®^ tool was developed to guide patients to ask three questions during healthcare visits to better understand their health conditions: What is my main problem? What do I need to do? Why is it important for me to do this? (Institute for Healthcare Improvement (IHI), n.d.). The Tell‐Us Card was developed to elicit patient preferences and participation in daily care in hospital settings (van Belle et al., 2020; Jangland et al., 2012). Patients are invited to communicate to healthcare providers what is important for them about their care each day during their hospital stay (van Belle et al., 2020; Jangland et al., 2012). Such tools have shown improved patient participation in decisions about care (van Belle et al., 2020; Jangland et al., 2012), and they have facilitated communication with healthcare providers (Lapiz‐Bluhm et al., 2015; Smith, 2020). However, a gap remains in the development and implementation of communication tools to support resident and family engagement in care conversations and planning with the care team in LTC. Including user perspectives is an important step to the development of tailored, patient‐focused communication tools (Clochesy et al., 2015). Research highlights the importance of including patients and their carers’ in research activities to better meet their needs and improve health outcomes (Manafo et al., 2018).

Prior to the current study, members of our research team (RM and SH) conducted focus groups with families about their care experiences, training, and education needs, to optimise their involvement as care partners in LTC. Families expressed a need for training and skills in how to give and receive information, and how to communicate with the care team more effectively (S. Helfenbaum, R. Meyer et al., unpublished data, 2018). Information from the focus groups confirmed a need for a resident‐ and family‐centred communication tool in LTC and provided further rationale for this study.

The aim of this paper is to describe the development of a communication tool for residents and families in LTC. The purpose of the tool is for residents and families to use as a guide for information sharing and to lead care discussions with the care team in LTC.

## 2 METHODS

We used a staged feedback approach to develop the communication tool. Our feedback approach was guided by Carman et al. (2013) Multidimensional Patient and Family Engagement Framework. There are three forms of patient engagement within their framework that range along a continuum including: Consultation, Involvement, and Partnership and Shared Leadership, with engagement occurring at different levels (i.e. direct care, organisational, policy‐making). Carman and colleagues highlight that although patient engagement is described on a continuum, one does not necessarily move along the continuum as a pathway. Rather, they describe how engagement is best approached as a range of opportunities aligned with the goal of engagement, as partnership and shared leadership may not be the aim. In this study, we were guided by the “consultation” form of engagement at the direct care level, as the goal of the engagement was to invite stakeholder input at different stages of development of the communication tool (Carman et al., 2013).

Our approach to tool development involved invited consultations with the following key stakeholders: our study collaborators (content experts), residents, and families (experts of their experience) (Kennedy, 2003). The study was conducted in Ontario, Canada. Ethics approval was obtained from the University of Toronto Health Sciences Research Ethics Board (#36220), and from our research team's study partners’ organisation. Operational approval was obtained from the two participating LTC homes. The tool development is part of our larger intervention study to test the feasibility and acceptability of the communication tool during resident‐ and family‐led huddles (scheduled brief meetings) with the care team.

### 2.1 Stage 1: Initial tool template development and consultation with the research team's study collaborators

Our research team developed and shared a draft template of the communication tool including an outline of its purpose with our study collaborators. Our three study collaborators have long standing relationships with members of our research team. These stakeholders have expertise and active roles in policy and in advocating for residents and family members in the LTC sector. Collaborators were emailed an invitation to provide written feedback on the communication tool template. The communication tool was developed to support resident and family communication with the care team in LTC. The tool is intended to be used by residents and their family member as a guide to organise information for them to lead a care discussion and share information proactively with the care team.

The draft tool template was based on best practices for engaging the patient and family to participate as partners in their care, such as actively seeking and clarifying patient and family goals, values, and needs in care planning (Toronto Academic Health Science Network Practice Committee University of Toronto Centre for Interprofessional Education & University Health Network, 2017). The initial communication tool template comprised six questions and several prompts intended to guide residents and their family member to reflect on and use for information sharing with the care team. For example, the first question in the original draft template asked: “Describe an important experience you had at the LTC home over the last month.” A resident might share an experience about a part of their care that they wish to have modified, such as receiving morning care later in the morning, so they can have a better sleep.

### 2.2 Stage 2: Consultation with family and residents

Following these initial stages to develop a communication tool template, we then conducted interviews with residents and families to engage them in further development of the tool to ensure the tool was tailored to meet their communication and information needs (Gonzales & Riek, 2013; Hoffman et al., 2020). Residents and family were recruited from two accredited LTC homes that had both a Resident and Family Council. To invite residents and family members to participate in an interview, we conducted information sessions at scheduled Resident Council meetings (attended by 4–6 members) and Family Council meetings (attended by 6–8 family members), arranged by the resident program director and family relations coordinator at each LTC home. Eligible residents were those who could communicate and speak English and provide written informed consent. Excluded from the study were residents with severe cognitive impairment (e.g. Cognitive Performance Score ≥4) (Canadian Institute for Health Information (CIHI) 2013). Residents who wrote their names down on the sign‐up sheet or verbally expressed interest were followed up with by the resident program director, and interviews for those interested were conducted at the beginning of their next scheduled Resident Council meeting.

Family members were eligible to participate if they could communicate and speak English and had a relative residing in a participating LTC home. An information sheet about the study including the research team contact information was distributed during these information sessions. A sign‐up sheet was also circulated during the information sessions for those interested in participating in an interview. Members of the team, the resident program director, and the family relations coordinator contacted interested family members to arrange the interviews. Two family members at one LTC home had agreed to provide feedback on the communication tool, and due to their conflicting schedules, individual interviews were arranged. Individual interviews were scheduled with family on an agreed upon time. A focus group was arranged for those interested at the beginning of their scheduled Family Council meeting.

Resident and family participants were a convenience sample of those who were present at the information sessions and had expressed an interest in participating and provided written informed consent. Written informed consent was obtained from resident and family participants by the lead investigator or by the trained project manager. Semi‐structured face‐to‐face individual interviews and one focus group interview were conducted during the spring of 2019. Interviews were conducted based on participant availability and were conducted during the day in a quiet area at the LTC homes by two research team members. Appendix S1 contains a checklist of reporting guidelines for interviews and focus groups (Tong et al., 2007). The tool was distributed at the beginning of the interviews. Residents and family members were asked to provide input and to share their perspectives about the communication tool's content and usability (e.g. appearance, ease of understanding) and to suggest any changes for improvement. At the beginning of the interviews, we described the purpose of the communication tool. The interview guide included questions such as: “Please comment on the language used in the tool;” “Do you have any suggestions for the wording?” and “What suggestions do you have for improving the content /information provided in the tool?” (Appendix S2). Interviews were not audio‐recorded as the research team (LC and GS) documented participant input directly on the tool in real time (Rutakumwa et al., 2020). Two research team members (LC and GS) conducted a content analysis of the data (Miles et al., 2015) after each round of feedback: family interviews, the focus group with family members, and resident interviews. Resident feedback was further compared with feedback from the family members to summarise the informational content. The analysis and subsequent changes to the tool were based on the participants' suggestions.

#### 2.2.1 Iterative approach to incorporating resident and family feedback

Individual interviews were conducted first with family members who were asked to share feedback on the tool, and they were invited to also provide written comments on the tool. Family members wrote their suggested changes directly on the tool during the conversation and submitted it to the research team member (LC) immediately after the interview. Feedback and proposed revisions from the family member interviews were incorporated into the tool. To garner a wider representative sample, a focus group was conducted with additional family members at the beginning of their scheduled council meeting. Revisions to the tool were made so that the family members in the focus group could see both the original version of the tool and the proposed revisions. Consistent with the patient engagement framework (Carman et al., 2013), after the family focus group meeting, the most refined version of the tool was shown to residents for feedback during individual interviews. We removed previous edits to make the tool more accessible to residents by reducing cognitive and sensory load.

## 3 RESULTS

The sample included a total of 11 participants. Two residents and two family members participated in individual interviews, 6 family members participated in one focus group, and one study collaborator (an executive in a non‐governmental organisation focused on family caregivers) provided written feedback. Residents were members of their LTC home's resident council. Residents did not have severe cognitive impairment but had high or very high care needs. Family participants were members of the LTC home's family council. All participants were English speaking. Feedback from the collaborator and that from resident and family interviews are described next, followed by integration of feedback to revise the tool and the benefits of engaging residents and family in research.

### 3.1 Initial tool template development: Feedback from the research team's study collaborator

Written feedback on the communication tool from the study collaborator resulted in two main suggestions (Table 1). First, the collaborator suggested clarifying that *“this tool can be used to lead a huddle about any issue or experience*.*”* Second, the collaborator highlighted the importance that the topic be prioritised in agreement by both the resident and family member. The collaborator indicated that: *“the resident and family member may disagree on which experience is the most important*.*”* We added a preamble (introduction to the tool) which was written at the top of the tool and incorporated the feedback from our study collaborator. These changes were made to the tool and shown to the residents and family during the interviews.

**TABLE 1:** Participant Role and Suggestions/Contributions for Revising the Communication Tool

Participant	Suggestions/Contributions
Study collaborator	Suggestions to enhance the use of the tool: clarify that the tool can be used to lead a huddle about any issue or experience the topic for discussion should be prioritised in agreement by both the resident and family member
Family members	Suggestions to enhance the tool's content and use of plain language: *Rephrase the language*: add the word significant to the opening question that asks to think about your most important experience revise the timeline to aid in recall—use the term lately rather than over the last month when asking to think about your most important experience add the word bothering to the end of the question that asks: Is the experience still affecting you? change the word support to help when asking: What support (if any) did you receive for this experience? change the word connect to talk when asking: Is there a staff/team member you would like to connect with about this? *Add questions*/*probes*: include a question/probe about a positive experience: What about the experience made you feel happy or good? add a question to the tool: How can the staff help you in the future? *Additional contributions:* remind residents to describe an experience here at the long‐term care home suggestions for potential application of the tool
Residents	Suggestions to enhance the tool's content and use of plain language: *Rephrase the language*: change the words to next time rather than in the future when asking: How can the staff help you in the future? add the word experience to the end of the question that asks: Is there a staff member you would like to talk with about this? *Add questions*/*probes*: add the following question (probe) to the last question in the tool: Are you looking for more information /resources? *Additional contributions:* suggestion for potential application of the tool

### 3.2 Feedback from family and resident interviews

Individual interviews lasted 15–20 minutes on average, and the focus group lasted 30‐minutes. Feedback from residents and family included suggestions to enhance the tool's content and use of plain language and suggestions for potential application of the tool (Table 1).

#### 3.2.1 Enhance the content and use of plain language

Family members suggested ways to make the preamble more informative. A family member suggested adding the word “significant” to the question we had included in the tool's preamble: “Think of your most important experience over the last month.” Another family member suggested that the timeline for reflection on the experience be revised to aid with recall. That is, rather than asking residents to think of an important or significant experience over the last month, the family member suggested the word “lately.”

Some of the wording used in the tool was rephrased to further tailor the language to residents and families. For example, the term “support” was considered too broad, and it was changed to a family member's suggested word “help” for ease of understanding. A family member suggested adding the word “bothering” to the end of the question that asked: Is the experience still affecting you? Some terms were further defined based on participant feedback. The notion that an experience could be positive was also highlighted in the tool, in response to a family member asking for examples of “what types of experiences.” This family member suggested that we include further questioning about the positive experience that asks the resident: “*What about the experience made you feel happy or good*?” Another family member suggested adding a question to the tool: “*How can the staff help you in the future?*” A resident further revised the timeline of this question (the word future) to “next time.” The last question in the tool was phrased as: Is there a team member you would like to connect with about this? If so, who? A family member suggested replacing the word “connect” to “talk” and further indicated that regarding naming the staff member to add: “*Would you like to mention who this person is*?”, while another family member indicated “*you don't need this part*.” One resident suggested adding the word “experience” to the end of the sentence: “*Is there a staff member you would like to talk with about this?*” This same resident suggested adding the following question (probe) to the last question in the tool: “*Are you looking for more information or resources?*”.

#### 3.2.2 Ease of use and application of the tool

All participants indicated that the communication tool was easy to use. One family member suggested for ease of use to remind residents to *“describe your experience here at the home*.*”* Participants described how the tool would help them to communicate and exchange information with the care team. Some participants shared specific suggestions for how they could personally apply the tool to improve a care situation or to reinforce positive relationships with the care team. For example, in providing feedback on the tool, one resident indicated that he would use the tool to discuss a current experience with requesting a team member to sign a document for him. A family member indicated that she would find the tool useful to help discuss her concerns about the provision of nutritional meals for residents at the LTC home. One family member suggested that if the experience discussed is a complaint *“a time limit could be used to follow*‐*up and streamline the process with the Ministry [Ontario Ministry of Health and Long*‐*Term Care] guidelines*.*”*

### 3.3 Integrating participant feedback

Following analysis of the interviews and the iterative revisions made to tool, our research team met to discuss the participant feedback overall. With regard to the final question in the tool, we left the question more general by removing the part about naming team members. During the huddles, 2–3 team members will be present and information sharing with additional team members can be discussed. Members of the research team suggested adding the words “impact you or your care” to the first question in the tool that asked about describing an experience to elicit further details. We used an existing communication framework called SBAR (Situation–Background–Assessment–Recommendation) as an organising schema for the tool's questions to further enhance its ease of use. We adapted the SBAR format to tailor the communication tool to be action‐ and outcomes‐oriented by replacing “Assessment” with “Actions,” and adding “Requests” after “Recommendations.” Formatting the communication tool using SBAR could further assist residents and families to organise information to communicate with team members and provide a structured, common language for discussions. The final tool comprised five main questions to guide the resident and family in care discussions with the care team (Appendix A1). The final version of the communication tool has a Flesch–Kincaid Grade 5 reading level (WebFX, 2020).

### 3.4 Benefits of engaging residents and family in research

Residents and families expressed their appreciation for the opportunity to provide feedback on the communication tool. They thanked us for allowing them to provide input and share their perspectives on the tool based on their own experiences communicating with the care team and the leaders (directors, managers) at the LTC home. This feedback process was beneficial for tailoring the communication tool for its intended users. We found that residents and families were engaged in the feedback process; they all fully participated, were supportive of each other's suggestions, and made valuable contributions to improve the tool's content and usability.

## 4 DISCUSSION

In this paper, we describe a staged feedback process that involved consultation with key stakeholders to develop a communication tool as part of a larger study. Feedback from the collaborator to provide more details about the topic for discussion resulted in incorporating a preamble to the tool for greater clarity. Further revisions of the tool were made based on residents’ and family members suggestions to enhance the tool's content and use of plain language. Potential uses of the tool were offered by participants. One suggestion for improving the tool was to include a recent timeframe for residents and family to reflect on an experience or situation to aid in recall. Outlining a clear timeframe within the tool's questions (where relevant) can aid in recall (Patton, 2014). Family and residents also suggested including additional content and more descriptive plain language in the tool. Ensuring the language is understandable to both residents and families can render the tool more usable and relevant for guiding care discussions with the care team (Stableford & Mettger, 2007). We also included positive language in the communication tool by indicating that the discussion could be focused on a positive experience. For example, if the resident and family described a team member's behaviour or action that was favourable and appreciated, this could be shared with the team so that they too could model this behaviour when appropriate and relevant. Indeed, unregulated care providers have reported that family members share concerns, but seldom offer praise (Majerovitz et al., 2009). Use of positive questioning can bring out the best in teams and supports collaborative practice (May et al., 2011) and a strengths‐based approach to care (emphasising a person's strengths and building on them) (Gottlieb, 2013).

To further enhance the tool's ease of use for communicating information to others (Renz et al., 2013) and in particular, for engaging residents and families in information sharing, we integrated SBAR language into the communication tool. SBAR is an evidence‐based communication tool that is used in healthcare to structure the sharing of relevant information among healthcare providers (Muller et al., 2018). A recent systematic review found studies that used an SBAR communication tool for patient handoffs in hospitals reported an improvement in patient safety outcomes, such as a reduction in the number of patient falls (Muller et al., 2018). The feasibility of using an SBAR communication tool has been demonstrated in LTC (Devereaux et al., 2016; Field et al., 2011; Renz et al., 2013). For example, the SBAR communication tool has been used in LTC settings for nurse–physician telephone communication and has had a positive impact on the quality of medication management (Field et al., 2011) and in reducing avoidable hospitalisations (Devereaux et al., 2016; Tena‐Nelson et al., 2012). SBAR could be used to enhance communication between patients and the care team (Clochesy et al., 2015; Denham, 2008). In LTC, use of a communication tool could empower residents to share their preferences for their care. Training residents and families to use SBAR communication could also address barriers to information exchange with the care team such as health literacy through the use of a common language and structure for communication (Denham, 2008; Stableford & Mettger, 2007).

Family and residents further suggested potential application of how the communication tool could be effectively used. Having a tool available as a guide is important to help residents and families share information to effectively communicate with the care team. The tool has potential to enhance care and outcomes through proactive information sharing, enhancing efficient communication, and building partnerships in the co‐production of care (McNeil et al., 2016; Scales et al., 2019).

Our study has several implications for future research. Engaging older adults, particularly those living with frailty or dementia, and family/care partners in healthcare research, is well‐recognised as a meaningful, value‐added activity (Bethell et al., 2018; Esmail et al., 2015; Holroyd‐Leduc et al., 2016; McNeil et al., 2016). Patient engagement in research involving care processes has been linked to positive effects such as empowering participants (Domecq et al., 2014; Esmail et al., 2015; Lavoie‐Tremblay et al., 2016) and giving them a voice in their healthcare (Holroyd‐Leduc et al., 2016), greater potential for applicability and uptake of research results to the target population (Domecq et al., 2014; Esmail et al., 2015), and potential for sustainable change (Baker et al., 2016; Castro et al., 2018). Although there are important considerations for engaging vulnerable older adults in research, such as cognitive impairment (Holroyd‐Leduc et al., 2016), patient engagement involves various levels of engagement and activities (Carman et al., 2013) where they can make valuable contributions and demonstrate their capabilities (Bethell et al., 2018).

Our findings present initial steps towards research that seeks to create engagement‐capable environments (Baker et al., 2016). At an organisational level, consulting key stakeholders on the development (and subsequent testing) of a tool could be one strategy for creating structures and processes for an engagement‐capable environment (Baker et al., 2016). Engagement‐capable environments are organisations that are successful in creating and sustaining a culture of patient‐centredness and engagement (Baker et al., 2016; Rowland et al., 2018). They have the necessary infrastructure and supports in place (e.g. effective leadership, clearly defined roles for patients) to encourage and embrace ongoing meaningful engagement of patients and families/care partners with the care team and leaders to deliver high‐quality care (Baker et al., 2016; Rowland et al., 2018). Residents and families who are willing and able to be active partners in care require skill‐building and support (Nickel et al., 2018; Scales et al., 2019) through deliberative practice (International Nursing Association for Clinical Simulation and Learning (INACSL), 2016). We are piloting the communication tool virtually in our larger study and providing training, practice sessions, and structured implementation through huddles led by residents and their family member and attended by 2–3 care team members.

### 4.1 Strengths and limitations

A strength of this study was the inclusion of residents and family for their input and feedback on the communication tool. Resident and family input provides an initial evidence‐base for application of the tool for practice. The tool provides a common language that could improve the effectiveness of communication in care conversations (Shahid & Thomas, 2018), particularly for those living with dementia or other forms of cognitive impairment. The communication tool also has implications for including personal support workers in team communication. There are also limitations to note. Feedback was provided by one study collaborator, and feedback from additional collaborators may have led to further revisions of the tool at this development stage. Interviews were conducted with only two residents who may or may not be interested in participating in the implementation of the tool. Future studies seeking resident feedback should include a larger sample. Future research could explore how many voices are sufficient for patient engagement studies where feedback is sought. The sample included only those who speak English, and the communication tool is offered in English only. In our future work, providing translated versions of the tool will be explored. Another limitation is that the perspectives of the care team (e.g. nurses, personal support workers, allied healthcare providers) are not represented in this stage of tool development; however, their feedback will be sought during testing of the tool in our larger study. Future research is needed to understand how this tool could be used when residents and families have different needs. Research should examine the impact of patient engagement on the uptake of tools and interventions for quality improvement.

## 5 CONCLUSION

The communication tool developed offers a structured format to support and value the participation of residents and families in information sharing with the care team. It creates an opportunity to empower residents and family to lead care conversations and personalise resident care. Feedback highlighted the value of engaging residents and family in the development of a communication tool to enhance information sharing. The tool could facilitate the creation of communication structures and processes or further enhance those already established, to achieve quality and safety through collaborative practices.

## CONFLICT OF INTEREST

The authors declare that they have no competing interests.

## AUTHOR CONTRIBUTIONS

LC, WD, LMH, and KM designed the study. SH and RM provided data that informed the tool template. LC and GS conducted the interviews and analysis. SH and DG provided overall guidance on tool development. GS provided project management. LC drafted the manuscript. All authors critically reviewed the manuscript. All authors read and approved the final manuscript.

## ETHICAL APPROVAL AND CONSENT TO PARTICIPATE

The study was approved by the University of Toronto Health Sciences Research Ethics Board (#36220) [initial approval June 22, 2018] and a site from our study partners. Written informed consent was obtained from all study participants.

## CONSENT FOR PUBLICATION

Not applicable.

## Supporting information

Appendix S1

Appendix S2

## Data Availability

No datasets are available from this study as it is outlined in the consent form that only the research team will have access to the data.

## APPENDIX A1

### COMMUNICATION TOOL

#### Huddle preparation

As residents and family members, you will be provided with this tool to help you reflect on any issue, concern, or experience before the huddle (for example, a positive experience or something you are concerned about). It is designed to help you highlight and prioritize the experience you would like to discuss with your care team. This tool will also help guide you step‐by‐step through the huddle (brief gathering). It is the resident's experience (discussed also with the family member).

#### Think of your most important/significant experience lately

1. *Situation*: Describe an experience that you had lately that impacted you or your care. Can you please share some details?
2. *Background*: In what ways did this experience affect you? Is the experience still affecting/ bothering you? [If a positive experience, what about the experience/event made you feel happy/good]
3. *Actions*: What help (if any) did you receive for this experience?
4. *Recommendations*: What are your ideas for us to work together? [How can the staff help you next time? What could be done differently?]
5. *Requests*: Is there a staff member you would like to talk with about this experience? Are you looking for more information/resources?
