# Supplementary material for: Development of communication tool for resident‐ and family‐led care discussions in long‐term care through patient and family engagement
Source: Int J Older People Nurs. 2021 Oct 7;17(2):e12429. doi: 10.1111/opn.12429 (PMC9285466; doi:10.1111/opn.12429)
Supplement: Supplementary file 1 — Appendix S1 [file OPN-17-0-s002.docx]

Supplementary File 2: Interview Question Guide

1. Please comment on the appearance of the communication tool (and readability, language used, easy to understand, etc).

Probe: What suggestions do you have for improving the appearance of the tool; do you have any suggestions for the wording?

2. Do you think this tool and its information would be useful during a huddle? If so, in what ways?

3. What suggestions do you have for improving the content /information provided in the tool?

4. What suggestions do you have for improving the tool’s relevance and usefulness for a huddle?

5. Do you have any other feedback or comments?
