# Supplementary material for: Development of communication tool for resident‐ and family‐led care discussions in long‐term care through patient and family engagement
Source: Int J Older People Nurs. 2021 Oct 7;17(2):e12429. doi: 10.1111/opn.12429 (PMC9285466; doi:10.1111/opn.12429)
Supplement: Supplementary file 2 — Appendix S2 [file OPN-17-0-s001.docx]

Supplementary File 1: COREQ 32-item checklist

Guide Questions/ Description

Item no/ Domain

| **Domain 1: Research team and reflexivity** |  |  |
| --- | --- | --- |
| Personal Characteristics |  |  |
| 1. | Interviewer/facilitator | Which author/s conducted the interview or focus group?  **The lead investigator (LC) and research manager (GS) conducted the interviews and focus group**. **(GS conducted one interview and co-facilitated the focus group).** |
| 2. | Credentials | What were the researcher's credentials? *E.g. PhD, MD*  **LC- PhD**  **GS- MA** |
| 3. | Occupation | What was their occupation at the time of the study?  **LC- Assistant Professor**  **GS- Research manager** |
| 4. | Gender | Was the researcher male or female?  **LC- female**  **GS- male** |
| 5. | Experience and training | What experience or training did the researcher have?  **LC- PhD in Nursing with experience conducting qualitative interviews with residents and family members in long-term care settings.**  **GS- MA in Sociology with experience conducting focus groups. Received training for this study by LC.** |
| Relationship with participants |  |  |
| 6. | Relationship established | Was a relationship established prior to study commencement?  **No relationship**. |
| 7. | Participant knowledge of the interviewer | What did the participants know about the researcher? e*.g. personal goals, reasons for doing the research*  **Reasons for doing the research were shared with participants prior to data collection.** |
| 8. | Interviewer characteristics | What characteristics were reported about the interviewer/facilitator? e.g. *Bias, assumptions, reasons and interests in the research topic*  **Our roles on the study were shared with participants**. **LC shared her interests in the research topic**. |

**Domain 2: Study design**

| Theoretical framework |  |  |
| --- | --- | --- |
| 9. | Methodological orientation and Theory | What methodological orientation was stated to underpin the study? *e.g. grounded theory, discourse analysis, ethnography, phenomenology, content analysis*  **A staged feedback approach guided the study. The study was guided by the Multidimensional Patient and Family Engagement Framework (Carman et al., 2013).** |
| Participant selection |  |  |
| 10. | Sampling | How were participants selected? *e.g. purposive, convenience, consecutive, snowball*  **We used a convenience sampling approach. The sample comprised those who were present at the information sessions, agreed to participate, and provided written informed consent.** |
| 11. | Method of approach | How were participants approached? e*.g. face-to-face, telephone, mail, email*  **Participants were approached through face-to-face information sessions**. |
| 12. | Sample size | How many participants were in the study?  **Participants included a total of 10 residents and family members who were interviewed, and one study collaborator (provided written feedback)**. **(N=11)** |
| 13. | Non-participation | How many people refused to participate or dropped out? Reasons?  **None**. |
| Setting |  |  |
| 14. | Setting of data collection | Where was the data collected? e*.g. home, clinic, workplace*  **Data were collected in two long-term care facilities**. **All interviews were conducted face-to-face**. |
| 15. | Presence of non-participants | Was anyone else present besides the participants and researchers?  **Non-participants were not present**. |
| 16. | Description of sample | What are the important characteristics of the sample? *e.g. demographic data, date*  **Residents were members of their facility’s resident council. Residents did not have severe cognitive impairment, but had high or very high care needs**. **Family participants were members of the facility’s family council**. **All participants were English speaking**. |
| Data collection |  |  |
| 17. | Interview guide | Were questions, prompts, guides provided by the authors? Was it pilot tested?  **An interview guide was used for data collection**. |
| 18. | Repeat interviews | Were repeat interviews carried out? If yes, how many?  **Repeat interviews were not conducted in this study**. |
| 19. | Audio/visual recording | Did the research use audio or visual recording to collect the data?  **No audio or visual recording was used for data collection**. |
| 20. | Field notes | Were field notes made during and/or after the interview or focus group?  **Notes were made during the interviews and focus group**. |
| 21. | Duration | What was the duration of the interviews or focus group?  **Individual interviews lasted on average 15-20 minutes and the focus group lasted 30 minutes**. |
| 22. | Data saturation | Was data saturation discussed?  **We used a staged feedback approach. Evaluation of the use of the communication tool will occur during implementation in the larger study**. |
| 23. | Transcripts returned | Were transcripts returned to participants for comment and/or correction?  **Not applicable**. |

**Domain 3: Analysis and findings**

| Data analysis |  |  |
| --- | --- | --- |
| 24. | Number of data coders | How many data coders coded the data?  **Two research team members (LC, GS) reviewed the suggestions from participants.** |
| 25. | Description of the coding tree | Did authors provide a description of the coding tree?  **Not applicable**. |
| 26. | Derivation of themes | Were themes identified in advance or derived from the data?  **The analysis and subsequent changes in the tool were based on stakeholders’ suggestions.** |
| 27. | Software | What software, if applicable, was used to manage the data?  **Not applicable**. |
| 28. | Participant checking | Did participants provide feedback on the findings?  **Participant checking was not conducted in this study**. |
| Reporting |  |  |
| 29. | Quotations presented | Were participant quotations presented to illustrate the themes / findings? Was each quotation identified? e*.g. participant number*  **Examples of participants’ suggestions for changes to the tool were presented in their own words in the manuscript.** |
| 30. | Data and findings consistent | Was there consistency between the data presented and the findings?  **Data and findings presented are consistent**. |
| 31. | Clarity of major themes | Were major themes clearly presented in the findings?  **Two main suggestions from participants’ feedback are presented in the results**. |
| 32. | Clarity of minor themes | Is there a description of diverse cases or discussion of minor themes?  **Not applicable**. |

Tong A, Sainsbury P, Craig J. Consolidated criteria for reporting qualitative research (COREQ): a 32-item checklist for interviews and focus groups. Int J Qual Health Care. 2007;19(6):349-357.
